# A risk- and needs-based strategy HIV prevention for adolescent girls and young women, WHO African Region

**DOI:** 10.2471/BLT.23.291160

**Published:** 2024-11-04

**Authors:** Sharana Mahomed, Elizabeth Bukusi, Izukanji Sikazwe, Philippa Musoke, Quarraisha Abdool Karim

**Affiliations:** a Centre for the AIDS Programme of Research in South Africa, Durban, South Africa.; b Kenya Medical Research Institute, Nairobi, Kenya.; c Centre of Infectious Disease Research in Zambia.; d Makerere University-Johns Hopkins University Research Collaboration, Kampala, Uganda.; e Centre for the AIDS Programme of Research in South Africa (CAPRISA), Doris Duke Medical Research Institute, University of KwaZulu Natal, 719 Umbilo Road, Durban 4001, South Africa.

The human immunodeficiency virus (HIV) epidemic in sub-Saharan Africa remains an important public health challenge, despite an estimated reduction of approximately 38% in global HIV incidence between 2010 and 2022.^1^ While antiretroviral therapy has increased life expectancy and reduced mother-to-child transmission, adolescent girls and young women aged 15–24 years continue to bear a disproportionate burden. In sub-Saharan Africa, the estimated HIV incidence rates among this population group range from about 1.5 to 5 per 100 woman-years, with prevalences up to eight times higher than for their male peers. Eastern and southern Africa are particularly affected, with adolescent girls and young women representing an estimated 62% of new HIV infections.^2^ Scale-up of antiretroviral therapy and efforts to reach the HIV-related 2030 targets have been crucial in increasing life expectancy and addressing onward transmission of HIV. However, in some settings, particularly in eastern and southern Africa, this progress has not translated into a significant reduction in new HIV infections.^3^ For instance, in countries like Eswatini, Lesotho and South Africa, despite high antiretroviral therapy coverage, HIV incidence rates remain concerning, especially among adolescent girls and young women.

In sub-Saharan Africa, where 65% of the population is younger than 35 years, the HIV epidemic is severely affecting adolescent girls and young women. The success of HIV prevention and treatment programmes has created a complex landscape for adolescent girls and young women, with three distinct groups requiring tailored interventions. These groups include HIV-exposed but uninfected adolescents, who may face unique health challenges related to living in high-prevalence communities and stigmatization, among others; perinatally infected individuals transitioning to adulthood, confronting issues such as treatment interruptions and sexual health management;^4^ and adolescent girls and young women living with sexually acquired HIV, who face distinct barriers to care. Barriers may depend on geographical location, socioeconomic status and available support networks, and are unique to this group due to age-specific stigma, psychosocial and developmental factors, lack of youth-friendly health-care services, and legal and social barriers. In 2021, about half of this group achieved viral suppression compared to almost three quarters in older HIV-positive individuals, heightening the risk of secondary transmission and adverse maternal outcomes including postpartum death.^5^

This convergence of vertical and horizontal transmission of HIV threatens two decades of progress in the prevention of mother-to-child transmissions, the goal of eliminating these transmissions, and the United Nations 2030 goal of ending acquired immunodeficiency disease syndrome as a public health threat. Addressing the challenge of new HIV infections among this population demands a paradigm shift that transcends current approaches to HIV treatment and prevention. By considering the way in which HIV risk facing adolescent girls and young women is cumulative over time, the global health community can develop more effective, targeted interventions that integrate HIV prevention with comprehensive sexual and reproductive health services. Doing so has the potential to longitudinally prevent HIV acquisition and transmission and promote overall health and well-being for adolescent girls and young women. Additionally, new cohorts of infants will have the possibility of being born HIV-free, and reaching adulthood and their full potential as healthy individuals.

## Cumulative HIV-risk for adolescent girls and young women

### Preconception and early childhood

The risk of acquiring HIV begins even before conception. For women living with HIV, preconception counselling and care play a crucial role in preventing mother-to-child transmission and ensuring the health of mother and child. The use of antiretroviral therapy during pregnancy and breastfeeding have dramatically reduced transmission rates from 30–40% in the 1990s and early 2000s to less than 2% in 2015,^2^ demonstrating the effectiveness of targeted interventions during the first 1000 days of life.^6^ However, the impact of HIV extends beyond transmission. Even uninfected children born to HIV-positive mothers may face long-term effects from in utero HIV exposure, potentially affecting their development and health outcomes. Early diagnosis of HIV in infants and young children remains a significant challenge, often delaying crucial treatment initiation. This delay, coupled with HIV’s profound impact on childhood nutrition and growth, necessitates a comprehensive approach that extends beyond mere viral suppression.

### School-age years

The school-age years present a critical opportunity for HIV education and prevention. School-based HIV education programmes play a vital role in building life, resilience and coping skills as well as knowledge that can protect children as they grow. However, these efforts must also address HIV-related stigma in educational settings, which can affect the well-being and educational outcomes of affected children. The challenge lies in creating inclusive environments that support all children while providing targeted assistance to those affected by HIV.

### Adolescence

As young girls transition into adolescence, they face a confluence of factors that dramatically increase their HIV risk. Age-disparate relationships where young women acquire HIV from male partners 8 years older or more are a key driver of new infections. Socioeconomic inequalities limit the agency of adolescent girls and young women in sexual relationships, increasing their exposure to HIV. Limited access to education and economic opportunities often leads to early sexual activity and transactional sex as survival strategies. This exposure to older, HIV-positive partners, combined with limited ability to negotiate safe sex, increases their risk. Cultural practices, such as early marriage and gender-based violence, further amplify these vulnerabilities, while HIV-related stigma hinders adolescent girls and young women’s access to essential prevention and treatment services. Several biological factors such as genital inflammation and co-infections with sexually transmitted infections enhance HIV acquisition rates in this population group.^7^ Mental health challenges specific to adolescent girls living with HIV, coupled with the impact of substance use, create additional layers of vulnerability.

### Young adulthood

As adolescent girls and young women enter young adulthood, they face evolving challenges in HIV prevention and care. Challenges in HIV disclosure within relationships can complicate prevention efforts and adherence to treatment. Additionally, the impact of migration and urbanization on HIV risk becomes more pronounced, as young women navigate new social and economic landscapes. For adolescent girls living with HIV, adulthood brings concerns about long-term health complications, including an increased risk of certain cancers. The intersection of HIV with noncommunicable diseases presents new challenges for health-care systems and individuals alike. Furthermore, HIV can affect reproductive ageing, influencing family-planning decisions and health-care needs as women progress through different life stages.

### Pregnancy

HIV transmission is further sustained by low utilization of antenatal services among adolescent girls and young women, increasing the risk of mother-to-child transmissions. Undiagnosed and untreated HIV infection during pregnancy not only endangers the health of the mother but also increases the risk of vertical transmission, perpetuating HIV transmission across generations. For adolescent girls and young women living with HIV, these risks are even greater, particularly when viral suppression is not achieved.

## A strategy focused on the cumulative HIV risks and needs of adolescent girls and young women 

**Fig. 1:**
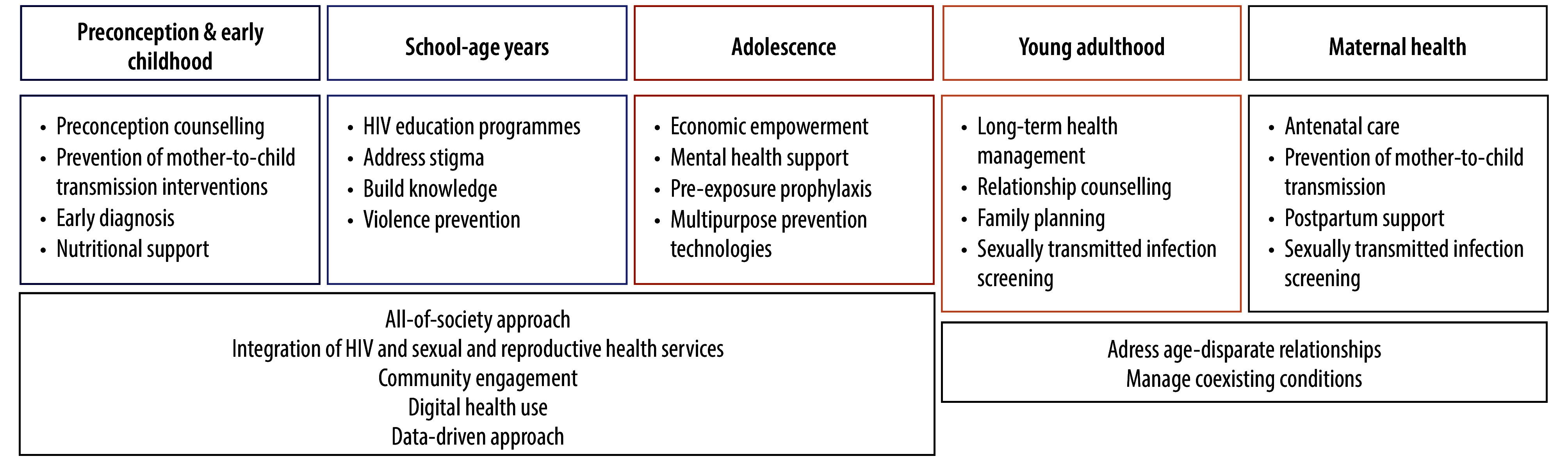
A strategy focused on the cumulative HIV risks and needs of adolescent girls and young women

The strategy we propose is tailored to address cumulative risks and needs related to HIV facing adolescent girls and young women across distinct life stages (Fig. 1). Central to this strategy is the seamless integration of HIV services with comprehensive sexual and reproductive health services, including family planning, sexually transmitted infections treatment, cervical cancer screening and mental health. This integration extends beyond health-care facilities, embracing an all-of-society approach that engages communities, schools, workplaces and policy-makers. The strategy uses lessons from global crises such as the coronavirus disease 2019 (COVID-19) pandemic to enhance service delivery and reach. Community-based service delivery, including mobile clinics and community health workers, has proven effective in maintaining access during health crises and reaching underserved populations.^8^

HIV: human immunodeficiency virus

An integral component is the incorporation of innovations in HIV prevention, particularly in pre-exposure prophylaxis and overcoming the low uptake and adherence challenges of oral pre-exposure prophylaxis among adolescent girls and young women. Long-acting injectables are promising options that could be an important complement to enhance programmes such as DREAMS, an HIV prevention programme that targets adolescent girls and young women particularly vulnerable to HIV.^9^ However, high costs and integration challenges may limit widespread access to these innovations, especially in low-resource settings. Ongoing innovations under evaluation include multipurpose prevention technologies^10^ that integrate antiretroviral drugs with contraceptive methods that could facilitate and enhance the multiple sexual and reproductive health needs of adolescent girls and young women in these settings.^11^

Challenges related to access, affordability and cultural acceptability will require innovative strategies to reach this group beyond traditional clinic-based models,^12^ which currently serve adolescent girls and young women who already use services. These strategies must target those disconnected from health systems. Social media and digital interventions offer new avenues for HIV prevention and have the potential to reach populations traditionally disconnected from health-care systems, providing information, support and linkages to care. Their implementation must be thoughtful and appropriate to the setting to ensure they address rather than widen existing health disparities.^13^ While promising, asymmetry in connectivity and access to health-care data for patients and health-care providers will need to be overcome to ensure digital solutions narrow rather than widen inequalities.

Strong and locally usable health information systems are crucial for monitoring and optimizing interventions aimed at adolescent girls and young women while also evaluating their impact. This data-driven approach, combined with the integration of mental health support and economic empowerment programmes, provides a promising strategy to reduce HIV incidence and improve overall health outcomes among adolescent girls and young women. By incorporating digital innovations and integrating new prevention and treatment technologies, this comprehensive life-course strategy offers a context-specific path to transforming HIV care in sub-Saharan Africa. Cross-sector collaboration, policy reform and health-care adaptation are essential to its success and to better position adolescent girls, young women and a new generation of children to realize their full potential and live HIV-free. Although ambitious, this strategy represents a promising step towards ending the HIV epidemic among this population group by 2030. 
